# Intestinal Cellular Biomarkers of Mucosal Lesion Progression in Pediatric Celiac Disease

**DOI:** 10.3390/pharmaceutics13111971

**Published:** 2021-11-20

**Authors:** Serena Vitale, Mariantonia Maglio, Stefania Picascia, Ilaria Mottola, Erasmo Miele, Riccardo Troncone, Renata Auricchio, Carmen Gianfrani

**Affiliations:** 1 Institute of Biochemistry and Cell Biology-CNR, 80131 Naples, Italy; serena.vitale@ibbc.cnr.it (S.V.); stefania.picascia@ibbc.cnr.it (S.P.); ilaria.mottola@ibbc.cnr.it (I.M.); carmen.gianfrani@ibbc.cnr.it (C.G.); 2 Department of Translational Medicine & European Laboratory for the Investigation of Food-Induced Diseases, University Federico II, Via S. Pansini 5, 80131 Naples, Italy; mariantonia.maglio@unina.it (M.M.); erasmo.miele@unina.it (E.M.); troncone@unina.it (R.T.)

**Keywords:** pediatric celiac disease, biomarkers, villous atrophy, TCRγδ+ T cells, IL4, translational research

## Abstract

Celiac disease (CD) is a chronic intestinal inflammation caused by gluten ingestion in genetically predisposed individuals. Overt-CD and potential-CD are the two main forms of gluten intolerance in pediatric patients with different grades of intestinal mucosa lesion and clinical management. For overt-CD patients the gluten-free diet is mandatory, while for potential-CD the dietary therapy is recommended only for those subjects becoming clinically symptomatic overtime. To date, specific early biomarkers of evolution to villous atrophy in potential-CD are lacking. We recently observed an expansion of TCRγδ+ T cells and a concomitant disappearance of IL4-producing T cells in the intestinal mucosa of overt-CD patients compared to potential-CD children, suggesting the involvement of these two cells subsets in the transition from potential-CD to overt-CD. In this study, we demonstrated that the intestinal densities of IL4+ T cells inversely correlated with TCRγδ+ T cell expansion (*p* < 0.005) and with the serum levels of anti-tissue transglutaminase antibodies (*p* < 0.01). The changes of these two cell subsets strongly correlated with mucosal lesions, according to the histological Marsh classification, as the transition from M0 to M3 lesions was associated with a significant reduction of IL4+ T cells (M0 vs. M1 *p* < 0.04, M0 vs. M3 *p* < 0.007) and an increase of TCRγδ+ T cells (M0 vs. M1 *p* < 0.05, M0 vs. M3 *p* < 0.0006). These findings strongly suggest that the detection of TCRγδ+ and IL4+ T cells could serve as cellular biomarkers of mucosal lesion and targets of novel immunomodulatory therapies for CD.

## 1. Introduction

Celiac disease (CD) is a chronic intestinal inflammation caused by dysregulated immune response to dietary gluten in genetically predisposed individuals. The pathogenic mechanisms are multifactorial, and various environmental factors, other than gluten proteins, participate in its onset [1]. Genetic predisposition is determined by specific alleles of Human Leucocyte Antigen (HLA), such as DQA1*05-DQB1*02 (carried by DR3/DQ2.5 haplotype) and DQA1*03-DQB1*03 (carried by DR4/DQ8 haplotype) genes, that encode for DQ2.5 and DQ8 heterodimers, respectively. Several large population studies reported an association of CD with non-HLA risk alleles with different functions in the immune response, as genes implicated in the maturation and /or activation of T lymphocytes (CD28, CTLA4, ICOS, interleukin (IL)-2 and IL-21), [2,3]. CD can be diagnosed at any age and may present a broad spectrum of clinical manifestations. In particular, CD clinically can arise with gastrointestinal or extra-intestinal symptoms or being totally asymptomatic. In this latter case, the disease can be diagnosed in subjects screened for familiarity or by general population screening [4]. Despite the large spectrum of clinical manifestations, CD may present different grades of intestinal mucosa damage, ranging from a slight inflammation and normal villous architecture, as in potential CD (potential-CD), or a marked tissue inflammation with villous atrophy, as in overt-CD [5]. Potential-CD condition affects 10–15% of all CD cases, mainly in pediatric age. About one third of potential-CD patients develops the overt form with villous atrophy during an observational period of 9 years on a gluten-containing diet [6,7]. Whilst the only treatment for overt-CD is a lifelong gluten-free diet (GFD), for potential-CD the dietary treatment is recommended only to patients presenting symptoms [7].

A large body of studies have underlined the pathogenic role of gluten-reactive T lymphocytes carrying the T-cell receptor (TCR)-aß (TCRaß+ T cells), massively infiltrating the lamina propria of patients with overt-CD [8]. In particular, most of these T cells are clusters of differentiation cell surface molecules 4+ (CD4+) TCRaß+ cells which recognize gluten peptides complexed with HLA-DQ2/DQ8 molecules and produce a cascade of inflammatory Th1 cytokines, dominated by interferon(IFN)-γ and interleukin(IL)-21 [8,9,10,11]. Moreover, the massive expansion of lymphocytes in the intestinal epithelium (intraepithelial lymphocytes-IELs) is a hallmark of CD, particularly of the overt disease [12]. The IEL compartment is constituted by CD8aß+TCRaß+ T cells, that express activating natural killer (NK) cell receptors (i.e., NKG2C and NKG2D) and kill stressed epithelial cells in a HLA-independent manner, and TCRγδ+ T cells, mainly CD4CD8-double negative cells, with an unclear antigen specificity and function [12,13,14]. The increase of TCRγδ+ IELs in untreated CD mucosa, their persistence in treated mucosa of patients on GFD, and the absence of this expansion in subjects with other small bowel enteropathies, suggest a specific relationship between their mucosa expansion and the pathogenesis of CD [13,14].

Recent evidence reports that both inflammatory CD4+ T cells reactive to gluten and immunoregulatory pathways are activated in the intestinal mucosa of potential-CD patients [9,15,16]. These findings suggest that an immune balance between pro-inflammatory and regulatory immune responses prevents the transition from normal mucosa to villous atrophy in those potential-CD patients who do not develop overt-CD over time. Nevertheless, the specific inflammatory pathways responsible for the progression of the intestinal lesion from mild to severe, as well as the regulatory mechanisms that contrast this transition from potential-CD to overt-CD, have not yet been clearly defined. We recently showed that in pediatric CD patients the intestinal mucosa with villous atrophy is characterized by an expansion of TCRγδ+ IELs and a concomitant disappearance of IL4-producing CD4+ T cells, if compared to the morphologically normal mucosa of potential-CD patients in which IL4+ cells were markedly present [17]. These IL4+ T cells showed two different cytokine profiles, as they co-produced or not INFγ and IL17, suggesting the presence of IL4+ cells with a Th2 phenotype. The IL4+INFγ- T cell subset, highly expanded in undamaged mucosa of potential-CD patients, suggest that this peculiar intestinal population may contrast the expansion/function of gluten-reactive Th1 cells producing IL21 and INFγ, and resulting progression of CD lesion from potential to overt-CD. Moreover, although regulatory T cells (Treg FoxP3+ and type 1 regulatory T cells) are also recruited in the intestine of overt-CD subjects [18,19,20], they fail to counterbalance the strong inflammatory response triggered by gluten [21], indicating that additional regulatory mechanisms might be involved to prevent the progression of duodenal damage in CD patients.

Our recent study [17] suggested that TCRγδ+ and IL4+ T cells could be cellular biomarkers of the evolution of mucosal lesion in CD. The identification of specific biomarkers, predicting the aggravation of tissue damage in subjects at the early stages of intestinal inflammation, could make it possible to discriminate, among the potential-CD patients, those at high-risk to develop the villous atrophy when on a gluten-containing diet. In order to address this point, we have further investigated these two T cell subsets in intestinal mucosa of CD patients with very mild mucosa inflammation (M0 Marsh score), slight inflammation (M1 Marsh score) or villous atrophy (M3 Marsh score). The intestinal frequency of TCRγδ+ and IL4+ T cells was also correlated with disease indices at the time of diagnosis, as anti-tTG2 immunoglobulin (Ig)A serum titres and mucosal lesion histological scores.

## 2. Materials and Methods

### 2.1. Study Population

The study population included a total of 55 young subjects followed at the Department of Translational Medical Sciences, Section of Pediatrics, Federico II, University of Naples: 19 children had overt-CD (mean age 6.1 years; range 1.9–11.7 years), 24 had potential-CD (mean age 9.3 years; range 1.1–17.4 years), and 12 were non-CD controls (mean age 6.3 years; range 1–13.9 years). Overt-CD and potential-CD patients had at the time of diagnosis positivity for the anti-tissue transglutaminase antibodies IgA (anti-tTG2 IgA, cut-off for positive serology: > 7 U/mL by immunoenzymatic assay and >30 U/mL by chemiluminescent immunoassay). Patients with clinical suspicion of CD underwent an esophago-gastro-duodenoscopy (EGDS). According to the Marsh-classification, 19 patients had overt-CD, with villous atrophy of small intestinal mucosa (Marsh score M3), 24 patients were classified as potential-CD. Of these, eight had a normal and not inflamed mucosa (Marsh score M0) and 16 had a mild lymphocyte infiltration, (Marsh score M1). Children with potential-CD were followed over time by the pediatricians to monitor clinical conditions and changes in the anti-tTG2 IgA antibody titres. All overt-CD patients were analysed at time of diagnosis and on a gluten-containing diet. The 12 children enrolled as non-CD controls underwent routine blood analysis and EGDS to exclude an organic disease or inflammatory condition, and all had a normal intestinal mucosa (grade Marsh 0/1). The *Helicobacter pylori* immunodeficiency and inflammatory bowel disease (IBD) were excluded after evaluation of laboratory parameters of inflammation, such as erythrocyte sedimentation rate (ESR), C-reactive protein (CRP) and fecal calprotectin (FCP). For this control group, the definitive diagnoses were: iron deficiency anemia, gastroesophageal reflux, recurrent abdominal pain, failure to thrive or nutritional deficiencies. Demographic, anti-TG2 titres, histological and clinical features of patients enrolled in the study were described in Table 1. Written informed consents to participate in the study were obtained from the patients themselves, or from parents if younger than 13 years. The study was approved by the Ethics Committee of the University of Naples “Federico II” (CE 383/20; CE308/16).

### 2.2. Immunohistochemistry

Immunohistochemical stainings were performed using at least four micrometer frozen duodenal sections. The number of cells expressing CD3 was determined in the intraepithelial compartment, with a value of CD3+ cells < 34/mm of the epithelium for M0, and >34/mm of the epithelium for Marsh score M1. Morphometric evaluations were performed using a microscope (Axioscop, Zeiss MicroImaging Inc., Milan, Italy) with a calibrated lens aligned parallel to the muscolaris mucosae.

### 2.3. Generation of Short Gliadin-Specific T Cell Lines (st-TCLs) for In Vitro Analysis

Gliadin-specific short-term T cell lines (st-TCLs) were generated from jejunal biopsies of eight overt-CD, 10 potential-CD and five non-CD controls. Biopsies were collected in RPMI-1640 medium (with 2% Human Serum-HS and 1.25 μg/mL amphotericin B deoxycholate, Lonza Group Ltd , Basel, Switzerland). Subsequently, the biopsy samples were digested with 1.5 mg/mL of collagenase-A from Clostridium hystolyticum (1 mg/mL; Roche, Mannheim, Germany), in 2 mL of culture medium RPMI-1640, supplemented with 1% penicillin/streptomycin antibiotics (Lonza Group Ltd), in incubation for 1 h and 30 min at 37 °C and 5% CO_2_ by stirring the plate every 15 min. After incubation with collagenase A, the cellular suspension was filtered through a 40 μm cell strainer filter (BD Falcon, Durham, NC, USA) and washed at 1500 rpm for 10 min. The cell viability and recovery were assessed by optical microscope assessed using trypan blue dye exclusion. The intestinal cells obtained were plated at approximately 5 × 10^5^ cells/well into a 24-well plate, in 1.5 mL of complete medium X-Vivo 15 with 5% HS and antibiotics penicillin (100 units/mL) and streptomycin (100 units/mL), and stimulated with 1.5 × 10^6^ irradiated (35 Gy = 3500 rad) autologous peripheral blood mononuclear (PBMC) cells, with 40 µg/mL native peptic-tryptic digest of gliadin (PT-G) and 40 µg/mL deamidated PT-G. The next day, 500 µL of complete medium was added supplemented with 20 U/mL IL2 and 5 ng/mL IL15 (R&D System Minneapolis, MN, USA) as growth factors, every 3 days. On days 7–9 and 21–24, st-TCLs were stimulated with irradiated autologous PBMCs and both forms of PT-G. After 21 days without further stimulation, the phenotype of st-TCLs was evaluated, while the intracytoplasmic cytokine production was detected after incubation with stimuli, both characterizations made by flow cytometry analysis, as follows. St-TCLs were incubated with or without phorbol 12-myristate 13-acetate (PMA, 10 ng/mL; CAS 16561-29-8-Calbiochem) and ionomycin calcium salt from *Streptomyces conglobatus* (1 µM) to stimulate cytokine production, and brefeldin A (10 µg/mL) from *Penicillium brefeldianum* (Sigma-Aldrich, St. Louis, MO, USA), was added to stop the cytokine secretion for intracytoplasmic stainings after total of 3-h stimulation. The phenotypic profile and cytokine production for the characterization of T-cell subsets were performed by multiparametric flow cytometric analysis as described below.

### 2.4. Intestinal Cell Isolation and Stimulation for Ex Vivo Analysis

Small intestinal biopsies collected for the ex vivo analysis were obtained from 11 overt-CD, 14 potential-CD and of 7 non-CD controls. The mucosal samples were digested with collagenase A from *Clostridium hystolyticum* as above described. At day 0, the phenotype of intestinal cells freshly isolated from both the epithelium and lamina propria was assessed by an ex vivo flow cytometric analysis. At day 1, intracytoplasmic cytokine production was evaluated after overnight incubation (ON) with 20 U/mL IL2 as growth factor, and subsequent mitogen stimulation (Cell Stimulation Cocktail, eBioscience, San Diego, CA, USA) for 3 h, as indicated by the manufacturers’ instruction.

Specifically, the freshly isolated intestinal cells were plated at cell density of 1 × 10^6^–1.4 × 10^6^ cells/well, in 24-well plates (Sarstedt AG & Co. KG, Nümbrecht, Germany), in complete culture medium with IL2 (20 U/mL, R& D System). After ON incubation, intestinal cells were stimulated by adding a mixture of PMA (stock 40.5 μM), ionomycin (stock 670 μM), brefeldin A (stock 5.3 mM), monesin (stock 1 mM) at the final dilution 1:500 (Cell Stimulation Cocktail, eBioscience) or unstimulated as negative control. Cells in suspension were harvested and stained for surface and intracytoplasmic staining, as described below.

### 2.5. Flow Cytometry

Intestinal cells (st-TCLs and freshly isolated from mucosal samples) were stained with the following fluorochrome labeled monoclonal (anti-human) antibodies: anti-CD3-Pacific Blue/-PerCP, anti-TCRγδ-FITC/-PE, anti-CD4-PECy7/-FITC, anti-CD8-APCCy7/-PE. Appropriate isotype-matched control monoclonal antibodies were included in all staining experiments. The intracellular cytokine staining was performed with fluorochrome-conjugated monoclonal antibodies: anti-IFN-γ-APC, anti-IL4 -PE. All analyses of cytokine producing cells were performed on both unstimulated and PMA/ionomycin-stimulated intestinal cells. All antibodies were purchased from BD Biosciences or Miltenyi Biotec (Bologna, Italy) and used at concentration according to the manufacturer’s instructions. Due to the small number of T cells infiltrating the intestinal biopsies, it was not always possible to analyze both the immunophenotype and the cytokine profile in the same patient. At least 1 × 10^5^ viable cells (assessed at microscope by trypan blue dye exclusion) were used for each staining done in phosphate saline (PBS)/0.5% bovine serum albumin (BSA) buffer. Surface staining of cells was carried out at 4 °C for 30 min. Intracellular staining was performed in permeabilization buffer (PBS/0.5% BSA with 0.5% saponin) on cells previously stimulated and fixed with 2% paraformaldehyde. Cells were next observed in the gate of viable mononuclear cells based on their forward-scatter/side-scatter characteristics. Samples were acquired with FACSCanto II and LSR2 flow cytometer supplied with BD FACSDiva software (Version 8.0, BD Biosciences, Milan, Italy).

### 2.6. Statistical Analysis

Statistical analysis was performed using a Mann-Whitney test by GraphPad Prism Software (Version 6, GraphPad Software, San Diego, CA, USA) to compare data between the groups, with a *p* ≤ 0.05 considered statistically significant. Correlations between variables were assessed using the non-parametric Spearman’s rank correlation coefficient by GraphPad Prism Software, with a *p* ≤ 0.05 considered as statistically significant.

## 3. Results

### 3.1. TCRγδ+ T Cells and IL4+ T Cells Are Inversely Correlated in Small Intestinal Mucosa

**Figure 1:**
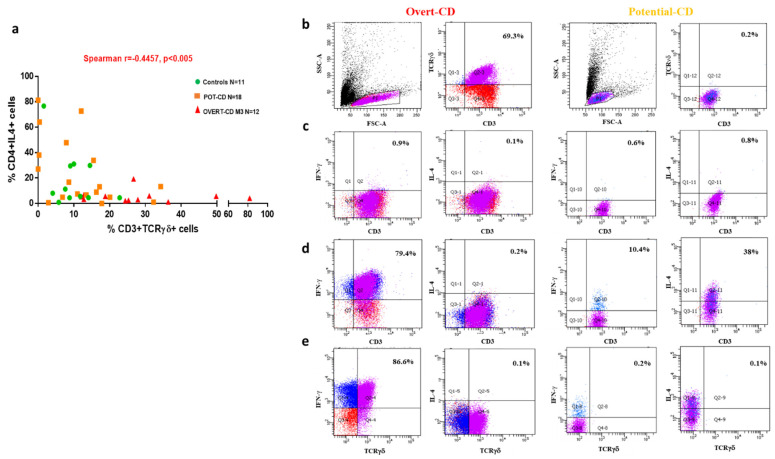
Correlation between the frequency of TCRγδ+ T cells and IL4+ T cells in potential- and overt-CD patients. (**a**) The percentages of TCRγδ+ T cells and IL4+ T cells were measured in duodenal mucosa samples of 41 children enrolled in the study (*n* = 12 with overt-CD, 18 with potential-CD and 11 non-CD controls). (**b**) The densities of CD3+ and TCRγδ+ cells were analyzed by a multiparametric flow cytometry in unstimulated condition, whereas the INF-γ and IL4 production was assessed without (**c**) or with PMA/Ionomycin 3 h of stimulation (**d**,**e**) both on freshly isolated intestinal cells and on short-term CD3+ cell lines (st-TCLs), as detailed in the materials and methods. Each symbol indicates the cell percentages detected in each subject. The correlation was assessed using the non-parametric Spearman’s rank correlation coefficient by GraphPad Prism Software, with a *p* ≤ 0.05 considered statistically significant. Representative flow cytometry dot plots from one overt-CD (left panels of **b**–**e**) and one potential-CD (right panels of **b**–**e**) patient, showing the percentage of CD3+ and TCRγδ+ cells positive for IFNγ and IL4 production, are reported.

In this follow-up study, in order to corroborate previous findings [17], we investigated these two lymphocyte populations in a larger number of pediatric subjects including patients with overt-CD, potential-CD and an age-matched control group of non-celiac subjects (henceforth identified as controls). In particular, we investigated the frequency of TCRγδ+ and IL4+ intestinal T lymphocytes by a multiparametric flow cytometry analysis on both intestinal cells freshly isolated from the mucosal samples and short-term T-cell lines (st-TCLs). In order to increase the number of observations, the ex vivo data were pooled with short-term TCL data. A significant inverse correlation was observed between the percentages of TCRγδ+ and IL4+ T cells, in 41 children enrolled in the study (overt-CD *n* = 12; potential-CD *n* = 18; controls *n* = 11, Spearmean r = −0.4457, *p* = 0.0035), as shown in Figure 1a. In the undamaged intestinal mucosa of children with potential-CD or non-CD controls, high percentages of CD3+IL4+ cells (mean frequency value in potential-CD: 24.6%, range 0–72.7% and in controls: 18.8%, 0.9–76.6%), were associated with low frequencies of TCRγδ+ T cells (mean frequency value in potential-CD: 12.1%, 0–34.3% and in controls: 10%, 1.6–22.8%). Conversely, in CD mucosa, especially in the flattened intestine of overt-CD patients, elevated densities of TCRγδ+ T cells (30%, 12.1–81.7%), corresponded to low frequencies of IL4 producing T cells (5.4%, 1.2–19.3%). The gating strategy used to determine cell subsets densities is shown in Figure 1b–e.

We looked at the phenotype of st-TCLs in an unstimulated condition (Figure 1b) and the cytokine production profile before (Figure 1c) and after mitogen stimulation (Figure 1d,e). CD3+TCRγδ+ cells from overt-CD produced high levels of IFNγ but not IL4, as shown in left panels of Figure 1e. A similar production profile of CD3+TCRγδ+ cells was observed in potential-CD children, although this subset is poorly represented in potential-CD mucosa (right panels of Figure 1e).

### 3.2. Intestinal IFNγ+TCRγδ+ and IL4+ T Cells Are Markers of Mucosa Inflammation

**Figure 2:**
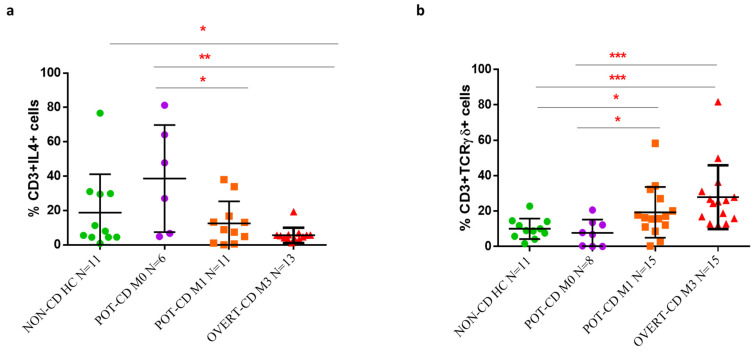
Frequency of TCRγδ+ T cells and IL4+ T cells in CD patients stratified on the base of intestinal inflammation degrees. (**a**) Percentages of IL4-producing T cells and (**b**) CD3+TCRγδ+ cells evaluated, by flow cytometric analysis, in intestinal mucosa (both ex vivo and in vitro approach) of non-CD controls, potential-CD with Marsh score M0, potential-CD with Marsh score M1, and overt-CD with villous atrophy (Marsh score M3). Data are shown as single data and mean and standard deviation of all experiments. Statistical analysis was performed using a Mann-Whitney test by GraphPad Prism Software, with a *p* ≤ 0.05 considered statistically significant and labelled with asterisk * *p* < 0.05, ** *p* < 0.01, *** *p* < 0.001.

To further examine the possible involvement of TCRγδ+ and IL4+ T cells in the progression of duodenal damage, the frequencies of TCRγδ+ and IL4+ T cells were calculated according to the histological Marsh-classification of the intestinal mucosa lesion, corresponding to M0 (Marsh type 0) in potential-CD children that show a morphologically normal mucosa with low lymphocyte infiltration, M1 lesion with lymphocyte infiltration, and M3 lesion typical of overt-CD patients with villous atrophy, in comparison to normal mucosa of non-CD control group. We observed that the changes of these T cell subsets density strongly correlated with mucosal lesions, as the transition from M0 to M3 was associated with a significant reduction of IL4+ T cells and an increase in TCRγδ+ T cells (Figure 2a,b). Specifically, a marked expansion of IL4+ T lymphocytes was observed in the group of potential-CD patients with M0 lesion (*n* = 6, mean frequency value 38.6%, range 4.9–81.3%) compared to potential-CD with M1 lesion (*n* = 11, 12.5%, 0.0–38%), and to overt-CD with M3 lesion that displayed the lowest cell infiltration (*n* = 13, 5.6%, 1.2–19.3%), (*p* = 0.03, M0 vs. M1 scored lesions; *p* = 0.0062 M0 vs. M3 scored lesions, Figure 2a). Similarly, a higher percentage of IL4+ T lymphocytes was found in normal mucosa of controls (*n* = 11, 18.8%, 0.8–76.6 %) compared to overt-CD with M3 lesion (*p* = 0.049, controls vs. M3, Figure 2a).

By contrast, an inverse trend was observed for the TCRγδ+ subset, as these cells were significantly less frequent in the subgroup of potential-CD with M0 lesion (*n* = 8, 7.7%, 0.0–20.6%) compared to the frequencies found in the subgroup with M1 lesion (*n* = 15, 19.3%, 0.3–58.3%), and overt-CD children with M3 lesion, this latter group showing the highest cell density (*n* = 15, 27.9%, 12–81.7%), (*p* = 0.04, M0 vs. M1; *p*= 0.0005, M0 vs. M3, Figure 2b). Lower percentage of TCRγδ+ T cells was measured in the control group (*n* = 11, 10%, 4.1–22.8%) in comparison to the percentage observed in potential-CD with M1 lesion and in overt-CD with M3 lesion (*p* = 0.03, controls vs. M1; *p* < 0.0001, controls vs. M3, Figure 2b). Although the TCRγδ+ and IL4+ cell densities differed between M1 potential-CD and M3 overt-CD, the difference did not reach a statistically significance (Figure 2a,b).

### 3.3. The Intestinal Densities of TCRγδ+ and IL4+ T Cells Correlate with Anti-Tissue Transglutaminase Serum Titres

The serum levels of anti-tTG2 IgA and anti-endomysial (EMA) antibodies have a high predictive value for the presence of gluten-dependent enteropathy in subjects with suspected CD [22,23]. Indeed, in case of high titres of anti-tTG2 IgA antibodies (≥10 times the upper limit of normal) and EMA IgA positive in a second blood sample, the histological evaluation of the intestinal mucosa damage is no longer necessary in pediatric patients, even without determining genetic predisposition and symptoms, according to recent guidelines [22,23].

**Figure 3:**
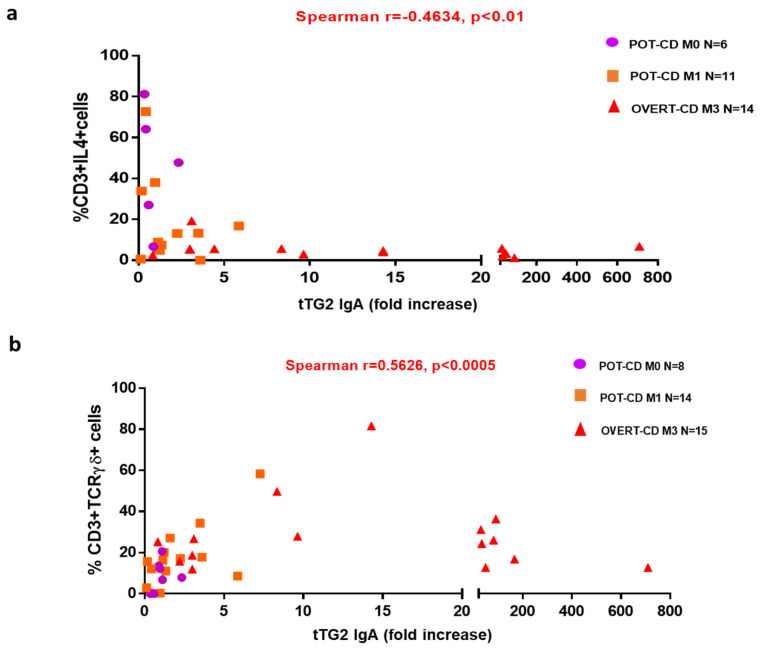
Correlation between the frequency of IL4+ T cells and TCRγδ+ T cells with serum levels of anti-tTG2-IgA antibody. (**a**) The correlation between the percentage of IL4+ T cells and (**b**) TCRγδ+ T cells and the serum anti-tTG2 levels was calculated in overall 37 CD patients grouped in potential-CD (M0), potential-CD (M1) and overt-CD (M3). The anti-tTG2 IgA levels (U/mL) were evaluated as fold increase respect to the specific threshold value indicated in the commercial kits, that were as follows: >7 U/mL by immunoenzymatic assay and >30 U/mL by chemiluminescent immunoassay. The correlation was assessed using the non-parametric Spearman’s rank correlation coefficient by GraphPad Prism Software, with a *p* ≤ 0.05 considered statistically significant.

However, children with a diagnosis of potential-CD may have a heterogeneous pattern of anti-tTG2 IgA antibody titres with values that can vary considerably during the clinical follow-up [6,7,24]. Based on the clinical and serological heterogeneity, we found worthy to investigate the possible correlations between the percentages of TCRγδ+ and IL4+ T cells and the serum anti-tTG2 titres, in CD patients enrolled in the study. The antibody titres were measured at the time of the evaluation of intestinal biopsy cell densities. Due to different commercial assays used for the determination of anti-tTG2 IgA levels in clinic, the antibody titres were evaluated as fold increase with respect to the threshold value provided by vendors (threshold titres for positive serology: tTG2 IgA > 7 U/mL by immunoenzymatic assay and tTG2 IgA > 30 U/mL by chemiluminescent immunoassay, as detailed in material and methods). A marked inverse correlation was observed among the frequencies of IL4+ T cells and the anti-tTG2 serum titres (r = −0.4634, *p* = 0.0087) in 31 CD patients, of which 17 were potential-CD (*n* = 6 M0, and *n* = 11 M1), and 14 were overt-CD (M3), (Figure 3a). By contrast, a significant direct correlation was found between the percentage of TCRγδ+ T cells and the anti-tTG2 serum levels (r = 0.5626, *p* = 0.0003), evaluated in 37 CD patients (*n* = 8 M0, *n* = 14 M1, *n* = 15 M3), (Figure 3b).

### 3.4. Serum Conversion of Potential-CD Patients Correlates with a High Frequency of Intestinal IL4+ T Cells

As previously mentioned, the anti-tTG2 IgA antibody titres can fluctuate over time in subjects with potential-CD, with a large variation during the clinical follow-up [24]. In some potential-CD subjects, even after several years, there might be a negative trend of anti-tTG2 IgA titres [6,7]. Some of potential-CD patients enrolled in the study were followed overtime (median value of follow-up years 2.7, range 1.3–8.5 years) and underwent more than one endoscopy. This allowed us to analyse for some potential celiacs the IL4+ and TCRγδ+ T cell percentages also at the time of serum conversion of anti-tTG2 titres. Although, we could not perform any longitudinal analysis of mucosal cells infiltration on those patients who underwent different endoscopies, we next separated the potential-CD children into four subgroups: (1) those who were seronegative for anti-tTG2 antibodies (with serum levels of anti-tTG2 IgA below the cut-off value) at the time of biopsy, (Seronegative POT-CD); (2) those who at time of our analysis were seropositive for anti-tTG2 antibodies but became seronegative (Seronegative POT-CD at follow-up); (3) those who remained seropositive during the follow-up, with serum levels above the specific cut-off (Seropositive POT-CD at follow-up); and (4) those that became overt-CD (Overt-CD at follow-up).

**Figure 4:**
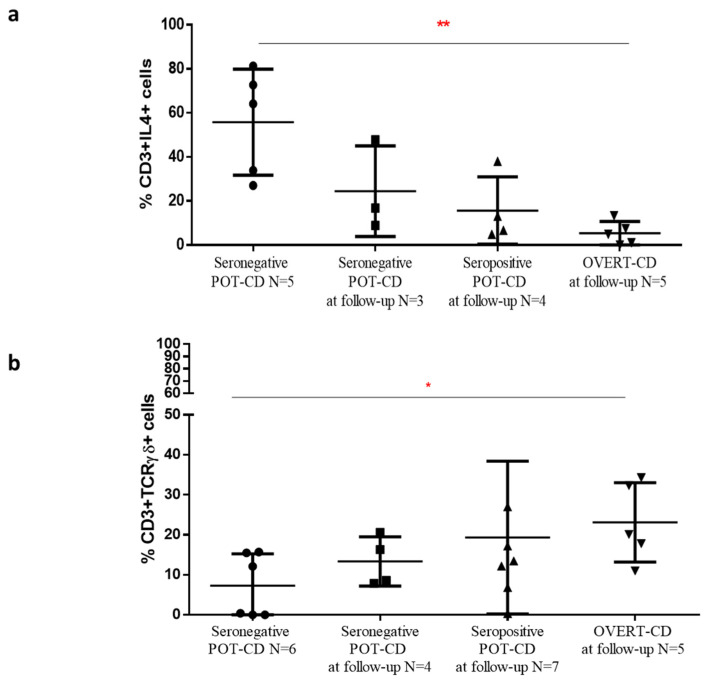
Densities of IL4+ T cells and TCRγδ+ T cells in potential-CD patients stratified on the base of the outcomes at clinical follow-up. The percentages of intestinal (**a**) IL4+ T cells and (**b**) TCRγδ+ T cells were calculated in duodenal samples of 22 children with potential-CD grouped on the basis of measurement of antibody titres and clinical evaluation done during the follow-ups, as follows: potential-CD with anti-tTG2 IgA levels below the cut-off at time of intestinal biopsy (Seronegative POT-CD); potential-CD with negative serology at follow-up (Seronegative POT-CD at follow-up); potential-CD with anti-tTG2 levels above the cut-off value at follow-up (Seropositive POT-CD at follow-up); potential-CD at time of flow cytometric analysis that developed villous atrophy over time, (OVERT-CD at follow-up). Data are shown as mean and standard deviation. Statistical analysis was performed using a Mann-Whitney test by GraphPad Prism Software, with a *p* ≤ 0.05 considered statistically significant and labelled with asterisk * *p* < 0.05, ** *p* < 0.01.

A higher percentage of IL4+ T cells was measured in the first patient group resulted negative at time of biopsy (55.8%, range 27–81.3%) compared to children that next developed overt-CD (5.3%, 0–13.3%, *p* = 0.008), (Figure 4a). The frequency of IL4+ cells found in this patient group was higher than the percentage observed in potential group that became seronegative during the follow-up (*n* = 3, 24.5%, 8.9–47.8%) and in potentials that were still positive (*n* = 4, 15.7%, 4.9–38%), although these differences did not reach a statistically significance (Figure 4a).

Instead, TCRγδ+ T lymphocytes showed an opposite trend, as they were significantly increased in potential-CD patients who developed overt-CD during follow-up (*n* = 6, 23.1%, 11–34.3%), compared to the percentage measured in potential-CD children that were seronegative at time of biopsy (*n* = 5, 7.3%, range 0–15.7%, *p* = 0.03), (Figure 4b). Likewise, a higher trend of TCRγδ+ T cell percentage was found in the group of potential-CD that remained seropositive (*n* = 7, 19.3%, 0.3–58.3%) and in the children with negative serology at clinical checks (*n* = 4, 13.4%, 7.9–20.6%), compared to potential-CD with negative serological values at the time of our analysis.

## 4. Discussion

CD may present with several forms, characterized by a large panel of clinical manifestations and enteropathy grades. The main CD forms are the overt disease, with total or partial villous atrophy, and potential disease, with a histologically normal mucosa. To date, it is non clearly understood if potential-CD is an intermediate condition before the evolution in overt disease. Furthermore, potential-CD represents a heterogeneous condition in which patients may further evolve to overt-CD by developing enteropathy and severe mucosa lesions, but also may reverse the pathological process and become CD-associated antibody seronegative over time [7,25].

Recently, we reported that the transition from a histologically normal intestinal mucosa of potential-celiacs to villous atrophy of overt-celiacs is associated with a marked intestinal expansion of TCRγδ+ T cells and a concomitant disappearance of IL4-producing T cells, likely CD4+ Th2 cells [17]. These preliminary findings strongly suggested that changes in the frequencies of these T cell populations in gut mucosa could influence the progression from mild towards severe mucosal inflammation and tissue damage. In the current follow-up study, we further analyzed the TCRγδ+ T cells and IL4+ T cells in gut biopsies of children with a diagnosis of potential- or overt-CD, in order to assess whether these two cell subsets could be cellular biomarkers of the intestinal damage progression in CD. In particular, a multiparametric flow cytometric analysis was performed to investigate frequencies, phenotype and cytokine production profile of mucosa infiltrating cells in the duodenal biopsies of a larger cohorts of children including cases with potential-CD, overt-CD, and age-matched non-CD controls. The above cellular parameters were correlated with serum anti-TG2 IgA antibody titres and mucosal lesion histological scores, according to Marsh classification. An indirect correlation between the frequency of these two cell subsets was observed in all children, including non-CD healthy controls. In the gut mucosa of overt-CD patients TCRγδ+ T cells were markedly present, in contrast to a low density IL4+ T cells, whilst an opposite cell distribution was observed in the biopsies of potential-CD patients and controls. In addition, TCRγδ+ T cells displayed a pro-inflammatory profile, as a great percentage produced IFNγ but not IL4, confirming published data showing a gluten-induced niche of inflammatory INFγ-producing TCRγδ+ IELs in damaged CD mucosa [14]. In potential-CD biopsies, in contrast to a very low percentage of TCRγδ+ T cells, we observed a consistent high densities of T lymphocytes producing IL4 and not IFNγ, most likely Th2 cells, but also T cells producing IL4 and IFNγ (Th0). Interestingly, these latter cells were more frequent compared to IFNγ-producing Th1 cells.

When the mucosa densities of TCRγδ+ and IL4+ T cells were correlated with the scores of mucosa damage, we found that the transition from M0 to M3 lesion was associated with a statistically significant reduction of IL4+ T cells and an increase of TCRγδ+ T cells. Of note, these significant differences in cell frequencies were observed also between potential-CD patients with M0 and M1 histology. In addition to already known expansion of IELs, of which TCR γδ+ cells represent an important component, we found a decreased frequency of IL4+ T cells in patients with M1 compared to M0 score, confirming that alteration in the percentage of these two T cell subsets are already measurable in the early-stages of inflammation, before the typical CD lesion occurs. Our findings have a relevance as, though in both M0 and M1 potential-CD conditions the villous and crypts architectures are normal, M1 score is characterized by an increased number of intraepithelial lymphocytes (CD3+ were >34/millimeter of the epithelium).

In our study, we observed a trend of increased percentage of IL4-producing T cells and decreased frequency of TCRγδ+ T cells in potential-CD with M1 stage compared to overt-CD with M3, although we did not find significant difference between the two CD patient groups. Recently, Ruiz-Ramírez et al. measured by flow cytometry our similar percentages of TCRγδ+ T cells in duodenal biopsies of a large and heterogeneous cohort of CD patients, both with M1 and M3 lesions, without finding differences between groups [26]. A prospective study on a large pediatric cohort with potential-CD, that performed multivariate analyses of clinical, genetic, and histologic data to identify factors associated with progression of mucosal damage, is in accordance with our results [7]. This study reported that potential-CD patients, who at the time of diagnosis were classified as M1, were more prone to develop villous atrophy compared to M0 potential-CD children. Furthermore, the higher number of TCR γδ+ T cells evaluated by immunohistochemical analysis on duodenal biopsies, was a risk factor for the progression of the intestinal lesion [7]. However, these interesting studies have not investigated the intestinal cell subsets producing IL4.

Given the high predictive value of elevated anti-tTG2 serum levels for the presence of a gut mucosa gluten-dependent lesion [22,23], we calculated the correlation between the anti-tTG2 IgA antibody titres and mucosa infiltrating T-cell populations of our interest. We demonstrated that CD-autoantibodies levels were indirectly correlated with IL4+ T cells, and directly correlated with TCRγδ+ T cells in all CD patients. These correlations strongly suggest that the combined detection of intestinal frequencies of TCRγδ+ and of IL4-producing T cells could be useful to predict the evolution to villous atrophy in potential-CD patients with low levels of anti-tTG2 IgA, in particular if slightly over the cut-off. In contrast to our findings, Ruiz-Ramírez et al. reported that the percentage of TCRγδ+ T cells is not influenced by the serum levels of anti-tTG2, although the high heterogeneity of their cohort of CD patients could explain this divergence [26].

A significant higher frequency of IL4+ T cells, concomitantly with a decreased percentage of TCRγδ+ T cells, was found in mucosal tissue of potential-CD patients that at the time of the flow cytometric analysis of intestinal samples were seronegative, compared to potential-CD that during the follow-up become overt-CD patients. These pilot results, if confirmed in larger cohorts, demonstrate that potential-CD is a mosaic disorder in which patients can fluctuate during clinical follow-up, and support the utility of the detection of these two cellular markers for management of potential-CD. To look at the densities of TCRγδ+ T cells and IL4+ T cells could help to classify potential-CD patients into those who will develop the villous atrophy from those who will reverse their disease condition, even on gluten containing diet.

Recent studies observed that the analysis of IELs flow cytometric pattern, including the increase of TCRγδ+ IELs, was an accurate and better method than anti-TG2 intestinal deposits for identifying CD in patients with lymphocytic enteritis, at the first diagnostic biopsy, also in seronegative subjects [27,28].

As mentioned, only a few studies investigated the role of IL4-producing T lymphocytes in the pathogenesis of CD, by providing data assessed with different methodical approaches, and often in contrast. Di Sabatino et al. measured, by cytokine array, the amount of the Th2 cytokines, as IL4, IL5, and IL13 in the supernatants of ex vivo-cultured duodenal biopsies taken from untreated and treated CD patients and from controls, without finding significant differences between the groups [29]. Similarly, Tiittanen et al., that in a previous study had reported an enhanced number of IL4+ cells in non-inflamed small intestinal biopsies of patients with Type 1 diabetes (T1D); [30], measured the expression of different cytokines, as IFN-γ, IL4, IL8, IL10, IL15 and IL18, by quantitative reverse transcription–polymerase chain reaction, in the small intestinal biopsies of pediatric patients with overt-CD and potential-CD, with comorbidity or not of T1D, and of control patients [31]. They did not found difference in mRNA expression of IL4, IL8 and IL15 between the children groups, unlike of IL10 mRNA expression observed higher in patients with overt-CD than in the other groups of subjects, and of IFN-γ mRNA increased in all CD patients with or without T1D [31]. These studies did not found difference in the quantity or m-RNA expression of IL4 in small intestinal tissues of CD patients and controls, although they did not evaluate the percentage of IL4-producing T cells but the total amount of the IL4 [29,31]. Conversely, in agreement with our results, another pediatric study demonstrated that in potential-CD children, with normal or slightly high percentage of intraepithelial TCRγδ+ T cells, a high percentage of IL4-producing cells, detected by immunohistochemistry, was present in the lamina propria [32]. The authors speculated a protective effect of this cytokine in potential-CD mucosa by down-regulating the inflammatory response, although in the study they observed higher densities of IL4+ cells in the gut of CD patients than in controls [32].

Whilst there is a unanimous accordance among clinicians on the necessity of a GFD therapy for patients with overt-CD, the management of patients with potential-CD is much debated, especially for the asymptomatic subjects. Thus, the identification of biomarkers, involved in the progression of the intestinal CD lesion, could be a useful approach to differentiate from the beginning the subgroup of potential-CD patients at high risk of developing overt-CD, in order to predict the transition from a normal to damaged mucosa, and to personalize the clinical treatment. A recent prospective study on a large pediatric cohort of CD-potential patients on gluten-containing diet, followed for up to 12 years, showed that about one third of children developed overt-CD and approximately one third of them became seronegative for anti-tTG2 antibodies [7]. Therefore, for some potential-CD subjects this phase is a first step towards the overt-CD form, and starting a preventive GFD may be necessary, while for others it is a transient pathological condition, and for these latter ones GFD may be an excessive treatment. In addition, it was demonstrated that, also for symptomatic potential-CD patients, symptoms and intestinal inflammation do not always improve after a long-term treatment with GFD [33].

In conclusion, we demonstrated TCRγδ+ and IL4+ T cells could be two biomarkers of the mucosal lesion evolution in CD, and their combined detection could represent a useful approach to better characterize the two CD phases, potential- and overt-CD. In addition, their detection in potential-CD patients at the first signs of intestinal inflammation, when the mucosal lesion has not yet advanced, could have a predictive value for the progression or remission of CD, and could be a clinical tool for the diagnosis and the management of these patients.

Although serological tests have a high specificity and sensitivity, the antibody titres can fluctuate, mainly in subjects with mild intestinal damage and a low gluten intake [34], in addition to the fact that there are many different kits on the market used for their detection. For these reasons, novel approaches for the diagnosis and the follow-up of CD are necessary. A recent paper, that analyses 49 relevant studies [28], concludes the flow cytometry ex vivo analysis of duodenal mucosal samples could be an accurate and powerful analytical tool for the diagnosis of CD. The management of potential CD is one of the most difficult challenges today. The identification of subjects at higher risk to develop the full blown disease would be very desirable. Our findings, in comparison to the studies reported in the [28], have evaluated the IL4-producing lymphocytes in addition to the TCRγδ+ intraepithelial lymphocytes, as potential cell biomarkers of CD lesion progression. The flow cytometry is currently a diagnostic strategy to evaluate minimal residual disease in several diseases such as acute leukemia or multiple myeloma. In the future, if our results are confirmed, the combined detection of these cell populations by flow cytometry could be an accurate and fast approach for diagnosis and monitoring of CD progression or remission. It would be also interesting to verify with further studies whether these cell biomarkers could be monitored also in peripheral blood, making their detection less invasive in comparison to an intestinal investigation, and providing clinicians of additional tools to identify among potential-CD patients those at high risk of progressing towards the overt-CD. When further studies will confirm the relevance of TCRγδ+ and IL4+ T cells as specific biomarkers of CD lesion evolution, their detection can be applied in clinical practice to assess the efficacy of new drugs for celiac disease treatment alternative to a gluten-free diet [35].

## Figures and Tables

**Table 1:** Clinical features of Caucasian pediatric subjects enrolled in the study.

Characteristics	Overt-CD	Potential-CD	Non-CD Controls
Patients	19	24	12
Gender (Male/Female)	8/11	4/20	5/7
Age (Mean years and range)	6.1 (1.9–11.7)	9.3 (1.1–17.4)	6.3 (1–13.9)
Anti-tTG2 IgA fold increase (Mean and range)	75.2 (0.83–709.3)	1.70 (0.17–5.86)	Levels below the cut-off value
Histology (Marsh score)	19 M3	16 M1, 8 M0	3 M1, 8 M0, 1 M3a

## Data Availability

On request.
